# Supplementary material for: Cannabis use, mental health, and problematic Internet use in Quebec: A study protocol
Source: PLoS One. 2024 Jun 3;19(6):e0304697. doi: 10.1371/journal.pone.0304697 (PMC11146692; doi:10.1371/journal.pone.0304697)
Supplement: S1 File — (PDF) [file pone.0304697.s001.pdf]

Centre intégré universitaire de santé et de services sociaux de l'Estrie – Centre hospitalier universitaire de Sherbrooke
Québec

Sherbrooke, le 14 juillet 2023

Pre Magaly Brodeur
FMSS
Université de Sherbrooke

Objet : Approbation finale du projet de recherche par le
Comité d'éthique de la recherche du CIUSSS de l'Estrie - CHUS

Projet #2024-5139 - CyberD-Cannabis
Consommation de cannabis à des fins non-médicales, santé mentale et cyberdépendance au Québec

---

Bonjour Pre Brodeur,

Le Comité d'éthique de la recherche du CIUSSS de l'Estrie - CHUS a pris connaissance en comité restreint de vos réponses et des documents suivants déposés via la plateforme Nagano à la suite de l'approbation conditionnelle du projet cité en rubrique.

Formulaire F20 #52857 déposé le 12 juillet 2023incluant :

- Protocole CyberD_2.0-2023-06-28.docx
- FIC Principal CyberD EN_1.0-2023-07-04.docx
- FIC en ligne CyberD ENG_1.0-2023-07-04.doc
- FIC Principal CyberD FR_2.0-2023-06-28.docx
- FIC En ligne CyberD FR_2.0-2023-06-28.docx
- Thèmes d'entrevue Phase 2 CyberD_1.0 -2023-06-29.docx
- Thèmes questionnaire Phase 1 - CyberD_1.0-2023-06-29.docx

Comme les réponses soumises et les modifications apportées à ces documents ont été jugées satisfaisantes, il nous fait plaisir de vous informer que votre projet a été **approuvé au plan éthique** par le CÉR du CIUSSS de l'Estrie - CHUS le **14 juillet 2023** et ce, pour une période de 12 mois, soit jusqu'au **14 juillet 2024.**

Liste des documents approuvés par le CÉR :

- Protocole CyberD_2.0-2023-06-28.docx
- FIC Principal CyberD EN_1.0-2023-07-04.docx
- FIC en ligne CyberD ENG_1.0-2023-07-04.doc
- FIC Principal CyberD FR_2.0-2023-06-28.docx
- FIC En ligne CyberD FR_2.0-2023-06-28.docx
- Thèmes d'entrevue Phase 2 CyberD_1.0 -2023-06-29.docx
- Thèmes questionnaire Phase 1 - CyberD_1.0-2023-06-29.docx
- Message type recrutement - CyberD1.0-2023-05-11.docx

Notes importantes:

SVP soumettre au CÉR le courriel de recrutement de la firme, le questionnaire (phase 1) et le texte des entretiens semi-dirigés (phase 2) lorsque vous serez prêts.

Seule la version finale du formulaire de consentement portant le sceau du CÉR devra être utilisée pour signature par les participants à la recherche (voir Nagano, onglet "Fichiers").

Vous devez attendre l'autorisation de la Direction de la coordination de la mission universitaire (DCMU) du CIUSSS de l'Estrie - CHUS avant de débuter la recherche. Certains aspects de la convenance organisationnelle doivent être évalués avant le début du recrutement des participants. Cette autorisation de la DCMU s'ajoute à l'approbation du Comité d'éthique.

Il est à noter qu'aucun membre du comité d'éthique participant à l'évaluation et à l'approbation de ce projet n'est impliqué dans celui-ci.

De plus, le CÉR confirme que vous avez déposé les documents requis pour établir que votre projet de recherche a fait l'objet d'un examen scientifique dont le résultat est positif.

En acceptant la présente lettre d'approbation finale du Comité d'éthique de la recherche du CIUSSS de l'Estrie - CHUS, vous vous engagez à soumettre au Comité:

- Toute demande de modification au projet de recherche ou à tout document approuvé par le comité pour la réalisation de votre projet.
- Seuls les incidents thérapeutiques graves ou les réactions indésirables graves (ITG/RIG) qui sont à la fois inattendus, possiblement reliés au produit de l'étude ET survenus chez un participant rattaché dans votre site **dans un délai de 15 jours de calendrier** suivant la prise de connaissance par l'équipe de recherche. Les événements ayant entraîné la mort d'un participant doivent être rapportés dans **un délai de 7 jours de calendrier**; (réf.: formulaire #F3 dans Nagano)
- Tout nouveau renseignement sur des éléments susceptibles d'affecter l'intégrité ou l'éthicité du projet de recherche ou d'accroître les risques et les inconvénients des sujets, de nuire au bon déroulement du projet ou d'avoir une incidence sur le désir d'un sujet de recherche de continuer sa participation au projet de recherche;
- Toute modification constatée au chapitre de l'équilibre clinique à la lumière des données recueillies;
- La cessation prématurée du projet de recherche, qu'elle soit temporaire ou permanente;
- Tout problème identifié par un tiers, lors d'une enquête, d'une surveillance ou d'une vérification interne ou externe;
- Toute suspension ou annulation de l'approbation octroyée par un organisme de subvention ou de réglementation;
- Toute procédure en cours de traitement d'une plainte ou d'une allégation de manquement à l'intégrité ou à l'éthique ainsi que des résultats de la procédure.

La présente décision peut être suspendue ou révoquée en cas de non-respect de ces exigences. En plus du suivi administratif d'usage, le CÉR pourra effectuer un suivi actif au besoin selon les modalités qu'il juge appropriées.

En terminant, nous vous rappelons que vous devez conserver pour une période d'au moins un an suivant la fin du projet, un répertoire distinct comprenant les noms, prénoms, coordonnées, date du début et de fin de la participation de chaque sujet de recherche.

Attestation du CÉR (REBA) :
En ce qui concerne ce projet de recherche, à titre de représentant du Comité d'éthique de la recherche du CIUSSS de l'Estrie - CHUS, je certifie que:

1. La composition de ce Comité d'éthique satisfait aux exigences pertinentes prévues dans le titre 5 de la partie C du Règlement sur les aliments et drogues.
2. Le Comité d'éthique de la recherche exerce ses activités de manière conforme aux bonnes pratiques cliniques.
3. Ce Comité d'éthique a examiné et approuvé le formulaire de consentement et le protocole d'essai clinique qui sera mené par le chercheur susmentionné, au lieu d'essai indiqué. L'approbation et les opinions du présent comité ont été consignées par écrit.
4. Ce Comité est conforme aux normes américaines. (FWA #00005894 et IRB #00003849)

Je vous prie d'accepter, Pre Brodeur, mes meilleures salutations.

Pour Me Johanne Obas,
Vice-présidente du CÉR du CIUSSS de l'Estrie - CHUS

Sonia Bachand

Mme Sonia Bachand
Bureau d'autorisations des projets de recherche
CIUSSS de l'Estrie - CHUS
Tél.: (819) 346-1110, poste 13861
sonia.bachand.ciussse-chus@ssss.gouv.qc.ca

Signé le 2023-07-14 à 12:06
