# Supplementary material for: Cannabis use, mental health, and problematic Internet use in Quebec: A study protocol
Source: PLoS One. 2024 Jun 3;19(6):e0304697. doi: 10.1371/journal.pone.0304697 (PMC11146692; doi:10.1371/journal.pone.0304697)
Supplement: S2 File — (PDF) [file pone.0304697.s002.pdf]

Sherbrooke, July 14, 2023

Pre Magaly Brodeur
FMSS
University of Sherbrooke

Subject: Final approval of the research project by the CIUSSS de l'Estrie - CHUS Research Ethics Board
Project #2024-5139 - CyberD-Cannabis
Non-medical cannabis use, mental health and cyberaddiction in Quebec

Hello Dr. Brodeur,
The Research Ethics Committee of the CIUSSS de l'Estrie - CHUS has reviewed your responses and the following documents submitted via the Nagano platform following conditional approval of the above-mentioned project.

Form F20 #52857 filed on July 12, 2023, including:

Protocole CyberD_2.0-2023-06-28.docx
CyberD Main FIC EN_1.0-2023-07-04.docx
CyberD Online FIC ENG_1.0-2023-07-04.doc
FIC Main CyberD FR_2.0-2023-06-28.docx
FIC Online CyberD FR_2.0-2023-06-28.docx
Interview themes Phase 2 CyberD_1.0 -2023-06-29.docx Questionnaire themes Phase 1 - CyberD_1.0-2023-06-29.docx

As the answers submitted and the modifications made to these documents have been deemed satisfactory, we are pleased to inform you that your project has been ethically approved by the CIUSSS de l'Estrie - CHUS REB on July 14, 2023, for a period of 12 months, until July 14, 2024.

List of documents approved by the REB:

Protocole CyberD_2.0-2023-06-28.docx
CyberD Main FIC EN_1.0-2023-07-04.docx
CyberD Online FIC ENG_1.0-2023-07-04.doc
FIC Main CyberD FR_2.0-2023-06-28.docx
FIC Online CyberD FR_2.0-2023-06-28.docx
Interview themes Phase 2 CyberD_1.0 -2023-06-29.docx
Questionnaire Themes Phase 1 - CyberD_1.0-2023-06-29.docx Recruitment Message Template - CyberD1.0-2023-05-11.docx

Important notes:

Please submit the firm's recruitment email, the questionnaire (phase 1) and the text of the semi-structured interviews (phase 2) to the REB when you are ready.

Only the final version of the consent form bearing the REB seal should be used for signature by research participants (see Nagano, "Files" tab).

You must wait for authorization from the Direction de la coordination de la mission universitaire (DCMU) of the CIUSSS de l'Estrie - CHUS before starting the research. Certain aspects of organizational suitability must be assessed before participant recruitment begins. This authorization from the DCMU is in addition to the approval of the Ethics Committee.

It should be noted that no member of the ethics committee involved in the evaluation and approval of this project is involved in this project.

Furthermore, the REB confirms that you have submitted the documents required to establish that your research project has undergone a positive scientific review.

By accepting this letter of final approval from the CIUSSS de l'Estrie - CHUS Research Ethics Board, you agree to submit to the Board:

- Any request for modification to the research project or to any document approved by the committee for the conduct of your project.
- Only serious adverse events (SAEs) or serious adverse reactions (SAIRs/SARs) that are unexpected, possibly related to the study product AND occurred in a participant attached to your site within 15 calendar days of the research team becoming aware of them. Events leading to the death of a participant must be reported within 7 calendar days (ref.: form #F3 in Nagano).
- Any new information on elements likely to affect the integrity or ethicality of the research project or to increase the risks and inconveniences to subjects, to interfere with the smooth running of the project or to affect a research subject's desire to continue participating in the research project.
- Any change in the clinical balance based on the data collected.
- Premature termination of the research project, whether temporary or permanent.
- Any problem identified by a third party during an internal or external investigation, monitoring or audit.
- Any suspension or cancellation of approval by a granting or regulatory body.
- Any procedure underway to deal with a complaint or allegation of a breach of integrity or ethics, as well as the results of the procedure.

This decision may be suspended or revoked in the event of non-compliance with these requirements. In addition to the usual administrative follow-up, the REB may carry out active follow-up as needed, according to the terms and conditions it deems appropriate.

In closing, we remind you that you must keep a separate record of each research subject's surname, first name, contact details and start and end dates of participation, for at least one year following the end of the project.

REB certification (REBA):

With respect to this research project, as representative of the Research Ethics Committee of the CIUSSS of Eastern Townships-CHUS, I hereby certify that:

1. The composition of this Ethics Committee meets the relevant requirements of Division 5 of Part C of the Food and Drug Regulations.
2. The Research Ethics Committee carries out its activities in accordance with good clinical practice.
3. This Ethics Committee has reviewed and approved the consent form and protocol for the clinical trial to be conducted by the above-mentioned investigator at the trial site.
investigator at the trial site indicated. The approval and opinions of this Committee have been recorded in writing.
4. This Committee complies with U.S. standards (FWA #00005894 and IRB #00003849).

Please accept, Pre Brodeur, my best regards.
For Maître Johanne Obas,

Mrs. Sonia Bachand
Vice-President of the CIUSSS de l'Estrie - CHUS REB

Research Project Authorization Office CIUSSS de l'Estrie - CHUS
Tel: (819)346-1110,ext13861 sonia.bachand.ciussse-chus@ssss.gouv.qc.ca

Signed on 2023-07-14 at 12:06 pm
