# Supplementary material for: Cannabis use, mental health, and problematic Internet use in Quebec: A study protocol
Source: PLoS One. 2024 Jun 3;19(6):e0304697. doi: 10.1371/journal.pone.0304697 (PMC11146692; doi:10.1371/journal.pone.0304697)
Supplement: Attachment — Submitted filename: CyberDCannabis_ResponseReviewers.docx [file pone.0304697.s003.docx]

Souparno Mitra

Academic Editor

***PLOS ONE***

March 25th, 2024

Dear Editors and Reviewers,

We are pleased to submit a revised version of our scoping review titled **“Cannabis use, mental health, and problematic internet use in Quebec (Canada): Study Protocol**” for publication in *PLOS ONE*. This protocol was co-authored by Magaly Brodeur, Didier Jutras-Aswad, Andrée-Anne Légaré, Adèle Morvannou, Eva Monson, Julie-Christine Cotton, Anders Hakansson, Virginie Parent, and Catherine Hudon.

We would like to thank you and the reviewers for your comments which allowed us to improve the quality of our manuscript. You will find attached a marked copy of the manuscript in which the changes are visible in track changes mode, as well as a clean copy. All typos have been corrected. You will find below our response *in italics* to each point raised by Reviewer 1 [R1] section by section of the article.

**Abstract**

- The authors said, “Problematic Internet use has been associated with cannabis use disorder, especially among adolescent users” in the Abstract. However, the present protocol excludes adolescents. Then, why do the authors want to emphasize the importance among adolescent users?

*Thank you for pointing this out. Our intention with this statement was not to emphasize the adolescent population but rather to point out there’s not a lot of evidence of this association other than in research on adolescent populations. The phrasing has been adjusted to express the lack of studies in the adult population instead.*

**Introduction**

- [R1] ll74-75, the statement “The COVID-19 pandemic has made problematic Internet use a priority issue for many…” needs more citation in addition to the current consensus statement. The following references may work.

Alimoradi, Z., Lotfi, A., Lin, C.-Y., Griffiths, M. D., & Pakpour, A. H. (2022). Estimation of behavioral addiction prevalence during COVID-19 pandemic: A systematic review and meta-analysis. Current Addiction Reports, 9, 486-517.

Ruckwongpatr, K., Paratthakonkun, C., Ghavifekr, S., Gan, W. Y., Tung, S. E. H., Nurmala, I., Nadhiroh, S. R., Pramukti, I., & Lin, C.-Y. (2022). Problematic Internet Use (PIU) in Youth: A Brief Literature Review of Selected Topics. Current Opinion in Behavioral Sciences, 46, 101150.

Chen, I.-H., Chen, C.-Y., Liu, C.-h., Ahorsu, D. K., Griffiths, M. D., Chen, Y.-P., Kuo, Y.-J., Lin, C.-Y., Pakpour, A. H., Wang, S.-M. (2021). Internet addiction and psychological distress among Chinese schoolchildren before and during the COVID-19 outbreak: A latent class analysis. Journal of Behavioral Addictions, 10(3), 731-746.

*Thank you for these suggestions. After examining the proposed references, we decided to cite the reference by Alimoradi et al. (2022) as it gathers a rich body of literature that supports our claim. The other two proposed references have been left out given they are either not specific to the COVID-19 pandemic context (Ruckwongpatr et al. (2022)) or focus exclusively on children (Chen et al. (2021)), while our study is interested in the adult population only.*

- [R1] Line 80, I think that (10,13,14) should be [10,13,14].

*This change has been made. Thank you.*

- [R1] When the authors propose their first objective (i.e., identify factors associated with problematic Internet use among cannabis users), it is unclear to me if this objective is based on hypotheses or is exploratory. This is important because if the objective will be investigated using hypotheses, the authors should provide theoretical background to support. However, if it is exploratory, the authors do not need to provide theoretical background. But even it is exploratory, the authors should provide some directions regarding what factors are of interests.

*Thank you for pointing this out. The objectives of our research remain exploratory. We have added a phrase to the first objective to provide more context as to what factors are of interest to us.*

**Methods**

- [R1] The authors want to recruit 1500 participants for the first phase of the present project, and the proposed sample size calculation is vague. Specifically, the authors simply said that “Sample size (n = 1500) was determined according to the number of participating individuals needed for the development of a regression model, composed of 10 variables associated with the epidemiological triad "host-agent-environment" [17] to determine the relationship between cannabis use and Internet use”. How exactly the sample size is calculated remains unclear to me.

*More information regarding how sample size was determined for this study has been added in lines 130 through 134.*

- [R1] Following the previous comment, I think that “sample size calculation” deserves to be a subsection instead of mentioning in the Data analysis section.

*Thank you for this suggestion. A subsection has been created for “sample size calculation” after the section “Recruitment and sampling.”*

- [R1] It is unclear to me how the authors can recruit the participants to be representative in terms of age, sex and gender, language, and geographic region. Say, what are the proposed sample size for each stratified group?

*A phrase has been added to the ‘Recruitment and sampling’ section to better explain how the stratified random sampling is applied to ensure representativeness.*

- [R1] Also, it is unclear how the representativeness of the database in the firm which will help distribute the questionnaire.

*A phrase has been added in the ‘Recruitment and sampling’ section stating that the web panels firm in charge of the recruitment has a respondent pool of over 200,000 members in Quebec, to which stratified random sampling is applied to ensure representativeness of this population.*

- [R1] As the data will be collected using two languages (i.e., English and French), I wonder if the authors will make sure the measurement invariance or check the linguistic validity between the two languages.

*It has been specified on lines 145 and 203-204 that validated questionnaires used for our data collection are available in both English and French, with the corresponding references provided.*

- [R1] The authors may consider using the ASSIST-11, a shortened ASSIST to assess other psychoactive substance use. The ASSIST-11 has been validated across 42 countries recently.

Lee, C. T., Lin, C. Y., Koós, M., Nagy, L., Kraus, S. W., Demetrovics, Z., Potenza, M. N., Ballester-Arnal, R., Batthyány, D., Bergeron, S., Billieux, J., Burkauskas, J., Cárdenas-López, G., Carvalho, J., Castro-Calvo, J., Chen, L., Ciocca, G., Corazza, O., Csako, R. I., Fernandez, D. P., … Bőthe, B. (2023). The eleven-item Alcohol, Smoking and Substance Involvement Screening Test (ASSIST-11): Cross-cultural psychometric evaluation across 42 countries. Journal of Psychiatric Research, 165, 16–27. <https://doi.org/10.1016/j.jpsychires.2023.06.033>

*Thank you for this suggestion, we will consider it for future projects. However, the protocol approved by the Research Ethics Committee and the institution providing funding for this research uses the 8-item ASSIST scale.*

- [R1] In the Phase 2 project, I would suggest the authors clearly mention the inclusion and exclusion criteria. Current presentation is not that clear. Also, I wonder if the authors will exclude those who have received treatments for cannabis use or internet use. Or, will the authors exclude those who have severe mental health disorder (e.g., schizophrenia)?

*Thank you for your comment. Phase 2 is a qualitative phase that aims to explore the experience of people with problematic Internet use (including those having sought treatment and/or diagnosed with a mental health disorder). We will therefore not exclude them from the interviews as we wish to study a plurality of experiences. To make the recruitment and sampling section clearer, for Phase 2, inclusion criteria has been numbered as such: “1) have participated in Phase 1 of the study, 2) have agreed to be contacted for Phase 2, and 3) present a problematic use of the Internet, according to IAT score during Phase 1, i.e., a score greater than 50”.*

- [R1] Lastly, I may overlook. However, the authors did not mention how and where they will deposit the data.

*Data will be stored on the Université de Sherbrooke's secure server and will be accessible upon reasonable request. A statement regarding data availability has been added to the article after the Acknowledgements section.*

This paper has not been submitted or published elsewhere, in part or in its entirety, and is not under consideration for publication elsewhere. All coauthors revised and approved the manuscript. We have read and understood your journal’s policies and have the following competing interests: Magaly Brodeur (MB) is a first-line physician and professor at Université de Sherbrooke. None of her research projects are funded by the gambling industry. She works as a prevention and harm reduction consultant for Loto-Quebec, the crown corporation responsible for state-run gambling in the province of Quebec (Canada).

Didier Jutras-Aswad (DJA) holds a clinical scientist career award from Fonds de Recherche du Québec (FRQS). DJA receives investigational products from Cardiol Therapeutics for a clinical trial funded by Quebec Ministry of Health and Social Services. This does not alter our adherence to PLOS ONE policies on sharing data and materials.

Thank you for your consideration. We look forward to hearing from you.

Sincerely,

**Magaly Brodeur MD PhD CCFP**

Université de Sherbrooke

Faculté de médecine et des sciences de la santé

Département de médecine de famille et de médecine d’urgence

3001, 12e Avenue Nord

Sherbrooke, Québec (Canada) J1H 5N4

Phone: (819) 821-8000 Extension 73349

Fax: (819) 564-5386

Email: [magaly.brodeur@usherbrooke.ca](mailto:magaly.brodeur@usherbrooke.ca)
