# Supplementary material for: Cannabis use, mental health, and problematic Internet use in Quebec: A study protocol
Source: PLoS One. 2024 Jun 3;19(6):e0304697. doi: 10.1371/journal.pone.0304697 (PMC11146692; doi:10.1371/journal.pone.0304697)
Supplement: Attachment — Submitted filename: CyberDCannabis_ResponseReviewers2.0.docx [file pone.0304697.s004.docx]

Souparno Mitra

Academic Editor

***PLOS ONE***

May 6^th^, 2024

Dear Editors and Reviewers,

We are pleased to resubmit a revised version of our scoping review titled **“Cannabis use, mental health, and problematic internet use in Quebec (Canada): Study Protocol**” for publication in *PLOS ONE*. This protocol was co-authored by Magaly Brodeur, Didier Jutras-Aswad, Andrée-Anne Légaré, Adèle Morvannou, Eva Monson, Julie-Christine Cotton, Anders Hakansson, Virginie Parent, and Catherine Hudon.

We would like to thank you and the reviewers for your comments which allowed us to improve the quality of our manuscript. You will find attached a marked copy of the manuscript in which the changes are visible in track changes mode, as well as a clean copy. All typos have been corrected. You will find below our response *in italics* to the point raised by Reviewer 1 [R1].

**Introduction**

- [R1] The authors have nicely responded to my previous comments and made great improvement. I only have one more minor suggestion. That is, the authors have cited Alimoradi et al.'s meta-analysis to support this statement, "The COVID-19 pandemic has made problematic Internet use a priority issue for many entities both in Quebec and abroad (government, researchers, health professionals, etc.), as it has led to increased use of the Internet and new technologies". I would suggest they further indicate that such internet use is associated with poor mental health as shown in another recent meta-analysis paper authored by the same team of Alimoradi.

Ref: Alimoradi, Z., Broström, A., Potenza, M. N., Lin, C.-Y., & Pakpour, A. H. (2024). Associations between behavioral addictions and mental health concerns during the COVID-19 pandemic: A systematic review and meta-analysis. Current Addiction Reports. <https://doi.org/10.1007/s40429-024-00555-1>

*Thank you for this suggestion. We have included a phrase in the introduction indicating the association between problematic Internet use and poor mental health, and its corresponding citation. Accordingly, the source has been added to the reference list.*

This paper has not been submitted or published elsewhere, in part or in its entirety, and is not under consideration for publication elsewhere. All coauthors revised and approved the manuscript. We have read and understood your journal’s policies and have the following competing interests: Magaly Brodeur (MB) is a first-line physician and professor at Université de Sherbrooke. None of her research projects are funded by the gambling industry. She works as a prevention and harm reduction consultant for Loto-Quebec, the crown corporation responsible for state-run gambling in the province of Quebec (Canada).

Didier Jutras-Aswad (DJA) holds a clinical scientist career award from Fonds de Recherche du Québec (FRQS). DJA receives investigational products from Cardiol Therapeutics for a clinical trial funded by Quebec Ministry of Health and Social Services. This does not alter our adherence to PLOS ONE policies on sharing data and materials.

Thank you for your consideration. We look forward to hearing from you.

Sincerely,

**Magaly Brodeur MD PhD CCFP**

Université de Sherbrooke

Faculté de médecine et des sciences de la santé

Département de médecine de famille et de médecine d’urgence

3001, 12e Avenue Nord

Sherbrooke, Québec (Canada) J1H 5N4

Phone: (819) 821-8000 Extension 73349

Fax: (819) 564-5386

Email: [magaly.brodeur@usherbrooke.ca](mailto:magaly.brodeur@usherbrooke.ca)
